# EB1-dependent long survival of glioblastoma-grafted mice with the oral tubulin-binder BAL101553 is associated with inhibition of tumor angiogenesis

**DOI:** 10.18632/oncotarget.27374

**Published:** 2020-02-25

**Authors:** Raphaël Bergès, Aurélie Tchoghandjian, Arnauld Sergé, Stéphane Honoré, Dominique Figarella-Branger, Felix Bachmann, Heidi A. Lane, Diane Braguer

**Affiliations:** ^1^ Aix-Marseille Univ, CNRS, INP, Inst Neurophysiopathol, Marseille, France; ^2^ Aix Marseille Univ, CNRS, INSERM, Institut Paoli-Calmettes, CRCM, Marseille, France; ^3^ APHM, CHU Timone, Marseille 13385, France; ^4^ Basilea Pharmaceutica International Ltd., Basel, Switzerland

**Keywords:** glioblastoma, cancer stem cells, microtubule-targeting agent, experimental cancer therapeutics

## Abstract

Glioblastoma (GBM) are aggressive brain tumors with limited treatment options. Cancer stem-like cells (CSLCs) contribute to GBM invasiveness, representing promising targets. BAL101553, a prodrug of BAL27862, is a novel small molecule tubulin-binding agent, promoting tumor cell death through spindle assembly checkpoint activation, which is currently in Phase 1/2a in advanced solid tumor patients including GBM. This study aimed to evaluate long-term daily oral BAL101553 treatment of mice orthotopically grafted with GBM CSLCs (GBM6) according to EB1 expression-level, and to decipher its mechanism of action on GBM stem cells. Oral treatment with BAL101553 for 100 days provoked a large EB1 expression level-dependent survival benefit, together with a decrease in tumor growth and brain invasion. Formation of vascular structures by the fluorescent GBM6-GFP-sh0 cells, mimicking endothelial vascular networks, was observed in the brains of control grafted mice. Following BAL101553 treatment, vessels were no longer detectable, suggesting inhibition of the endothelial trans-differentiation of GBM stem cells. *In vitro*, BAL27862 treatment resulted in a switch to the endothelial-like phenotype of GBM6 towards an astrocytic phenotype. Moreover, the drug inhibited secretion of VEGF, thus preventing normal endothelial cell migration activated by CSLCs. The decrease in VEGF secretion was confirmed in a human GBM explant following drug treatment. Altogether, our data first confirm the potential of EB1 expression as a response-predictive biomarker of BAL101553 in GBM we previously published and add new insights in BAL101553 long-term action by counteracting CSLCs mediated tumor angiogenesis. Our results strongly support BAL101553 clinical studies in GBM patients.

## INTRODUCTION

Glioblastoma (GBM) are the most frequent and aggressive primary brain tumors in adults with a median survival of only 15 months with the current standard of care [1]. The high heterogeneity due to functionally diverse cell types, hypervascularisation and the infiltrative nature of GBM tumor cells contributes to resistance to chemo-and radiotherapy [2, 3]. Cancer stem cells represent a subpopulation of cells within GBM that are characterized by increased resistance to chemotherapy and radiotherapy, suggesting that stem cells are likely responsible for failure of treatment and high recurrence rates [4-5]. Cancer stem cells are capable of infinite self-renewal and multi-potential differentiation [6]. Moreover, GBM stem cells can initiate very aggressive tumor formation when implanted in xenograft models. Therefore, GBM stem cells are considered as a relevant target for GBM therapy, and the elimination of these cells is crucial in treating GBM.

GBM stem cells have been reported to directly contribute to the tumor vasculature through trans-differentiation into endothelial cells [7-9]. GBM stem cell-derived endothelial cells possess the same vasculogenic activity as do endothelial cells [10, 11] and are able to organize into capillary-like tubes in 3D Matrigel cultures [12]. Furthermore, the connection between neural stem cells and the endothelial compartment seems to be critical in GBM, where cancer stem cells closely interact with the vascular niche and promote angiogenesis through the release of vascular endothelial growth factor a (VEGFa) and stromal-derived factor 1 [13, 14].

BAL27862 was previously shown to be a potent reversible microtubule (MT) destabilizer *in vitro*, when used at high concentrations, binding the colchicine site of the beta-tubulin [15]. It is a very potent inhibitor of tumor cell growth and promoter of cell death, whose activity is associated with activation of the spindle assembly checkpoint. Moreover, the drug is efficiently distributed into the brain and the tumor, with anticancer activity in GBM models, thus providing a strong rationale for its use for GBM treatment [16]. We recently demonstrated the anticancer activity of BAL27862 (*in vitro*) and its prodrug BAL101553 (xenografts) on the GBM cancer stem-like cell (CSLC) model GBM6. A survival advantage was observed after short-term intravenous treatment with indications of a reversal of GBM6 CSLC characteristics both *in vivo* and *in vitro* [16]. The A2B5^+^ GBM6 cell line was established from CSLCs isolated from a GBM patient. GBM6 cells display a mesenchymal phenotype with a high tumorigenicity and infiltrative pattern of migration *in vivo* [17, 18]. Moreover, we previously reported that microtubule (MT) +End-binding 1- protein (EB1) overexpression correlates with GBM progression and poor survival in a large cohort of GBM patients [19]. Importantly, the level of EB1 expression in GBM6 cells strongly influenced BAL27862/BAL101553 response (even at sub-cytotoxic concentrations *in vitro*) as drug treatment was less potent in EB1-downregulated GBM6 than in EB1-expressing control cells [16]. BAL101553, administered both orally and intravenously, is currently undergoing phase 1/2a clinical evaluation in patients with solid tumors including GBM.

This study aims to evaluate long-term daily oral BAL101553 treatment of mice orthotopically engrafted with GBM6, according to EB1 expression level, as well as to further decipher the mechanism of action of the drug on GBM stem cells. Daily oral treatment with BAL101553 (5 administrations a week for 100 days) provoked a dramatic survival benefit, together with a decrease in tumor growth and brain invasion, which was EB1 expression level dependent. Moreover, the drug counteracted tumor vessel formation in the brains of treated mice. *In vitro*, the drug switched the endothelial-like phenotype of CSLCs towards an astrocytic phenotype, and it inhibited secretion of VEGF; thus, preventing normal endothelial cell migration activated by CSLCs. Our study shows for the first time that BAL101553 treatment counteracts tumor angiogenesis by acting on CSLCs in an EB1-dependent manner and provides new insights into the therapeutic targeting of CSLCs. Altogether, our data strongly support clinical studies with long-term administration of BAL101553 in GBM patients.

## RESULTS

### Long-term daily oral BAL101553 treatment enhances survival, reduces tumor growth and invasion in mice orthotopically grafted with GBM6; an effect potentiated by EB1 expression

**Figure 1:**
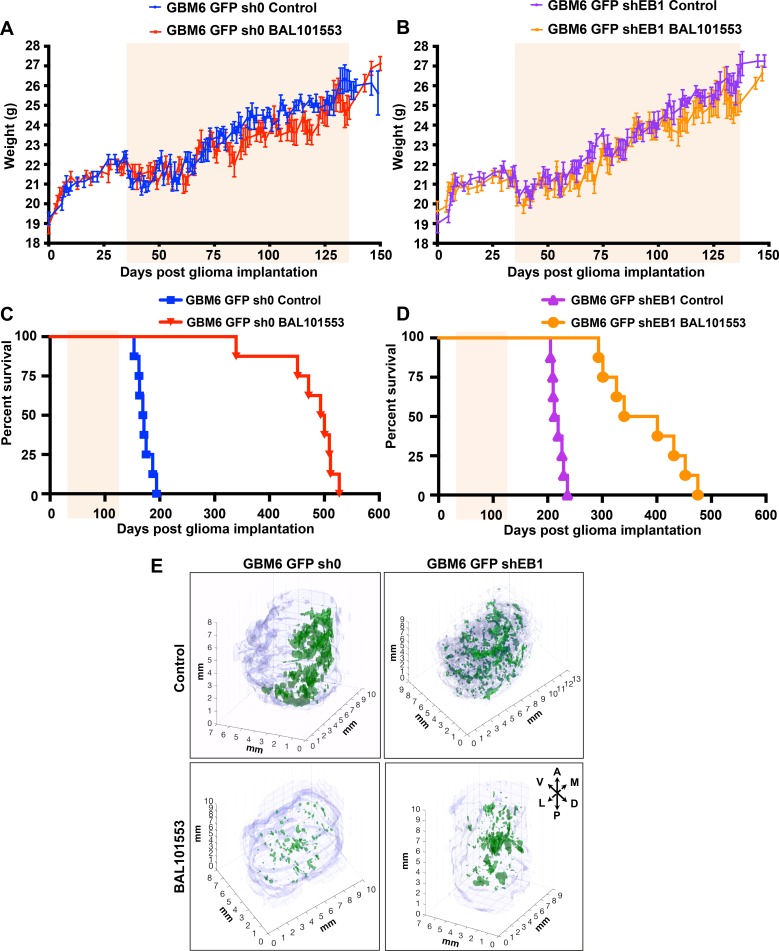
BAL101553 treatment enhances survival and reduces tumor growth in mice orthotopically grafted with GBM6 cells Mean weights of control GBM6-GFP-sh0 **(A)** or EB1-down-regulated GBM6-GFP-shEB1 bearing mice **(B)** orally treated with BAL101553 or vehicle for 100 days (pale orange square). Kaplan–Meier survival plot of GBM6-GFP-sh0 **(C)** or GBM6-GFP-shEB1 bearing mice **(D)**. Three-dimensional reconstruction of brains with tumors (green) (millimiter-scale). A, anterior; D, dorsal; L, lateral; M, medial; P, posterior; V, ventral **(E)**

To analyze long-term oral BAL101553 treatment *in vivo*, control GBM6-GFP-sh0 cells and EB1-downregulated GBM6-GFP-shEB1 cells were stereotaxically grafted into the subventricular zone of nude mice at day 0, and animals were orally treated with 30 mg/kg BAL101553 or vehicle control from day 35 to135 (5 administrations / week, 8 mice/group). Animals were monitored each day for weight loss, ataxia, and periorbital hemorrhage. Treatment was well-tolerated as evidenced by an absence of weight loss (Figure 1A and 1B) (Supplementary Figure 1A and 1B) or change in animal well-being as compared with vehicle-treated animals. When treated with vehicle, animals grafted with GBM6-GFP-shEB1 cells had prolonged survival time, compared to animals grafted with GBM6-GFP-sh0 cells (215.5 and 170 days for GBM6-GFP-shEB1 and GBM6-GFP-sh0 tumor-bearing animals, respectively) (Figure 1C and 1D, Table 1), confirming the bad prognostic value of EB1 (19). A significant long-term survival benefit was observed in the groups of animals treated with BAL101553. Indeed, median overall survival was extended by 326.5 days and 155 days for GBM6-GFP-sh0 and GBM6-GFP-shEB1 tumor-bearing animals, respectively, as compared with vehicle-treated animals. Moreover, death of tumor-grafted animals was observed at ages included in their normal life span [20]. Importantly, BAL101553 was more efficient if EB1 was not down-regulated (survival gain of 326.5 days, p<0.0001).

**Table 1:** Median survival of mice engrafted with control GBM6-GFP-sh0 or EB1-down-regulated GBM6-GFP-shEB1 cells and orally treated with BAL101553 or vehicle

Tumor	Treatment	n	Survival time (days)		Compared with vehicle	
			Range	Median	Survival gain (days)	p
GBM6-GFP-sh0	Vehicle (control)	8	162-187	170	-	-
GBM6-GFP-sh0	BAL101553	8	339-527	496.5	326.5	<0.0001
GBM6-GFP-shEB1	Vehicle (control)	8	209-236	215.5	-	-
GBM6-GFP-shEB1	BAL101553	8	293-475	370.5	155	<0.0001

Median survival time and statistical analysis of survival curves for control GBM6-GFP-sh0 or EB1-down-regulated GBM6-GFP-shEB1 and orally treated with BAL101553 or vehicle. The p values, calculated by the log-rank test, refer to differences between vehicle and BAL101553 treatment

Serial sagittal sections of the brains were collected at the end of drug treatment (135 days post-grafting) to analyze tumor invasion (Supplementary Figure 1C). Pictures taken from single sections were used for a 3D reconstruction of brains and quantification (Figure 1E). GBM6-GFP-sh0 cells from vehicle-treated mice largely invaded the brain. Indeed, large and invasive tumors were detected invading the cortex, the striatum and the contralateral hemisphere (Figure 1E top left panel, Supplementary Figure 1C, top left panel). In contrast, brain invasion was less pronounced in EB1 down-regulated tumor grafted mice (Figure 1E, top right panel, Supplementary Figure 1C, top right panel). Likewise, the volume of EB1-expressing tumor (sh0) was 7.1x10^-2^ mm^3^, while that of shEB1 tumor was only 1.9x10^-2^ mm^3^. Importantly, the volume of EB1-expressing tumor (sh0) after BAL101553 treatment was 1.6x10^-2^ mm^3^, resulting in a clear reduction of the tumor volume (-76%) and in tumor spreading indicating a suppressive effect of BAL101553 on tumor growth and brain invasion *in vivo* (Figure 1E, bottom left panel, Supplementary Figure 1C, bottom left panel). However, BAL101553 was less efficient (-48%) in shEB1 tumors (tumor volume of 1.0x10^-2^ mm^3^) as compared with vehicle-controls (Figure 1E, bottom right panel, Supplementary Figure 1C, bottom right panel).

### *In vivo*, BAL101553 induces loss of stem cell properties and inhibits tumor vasculature

**Figure 2:**
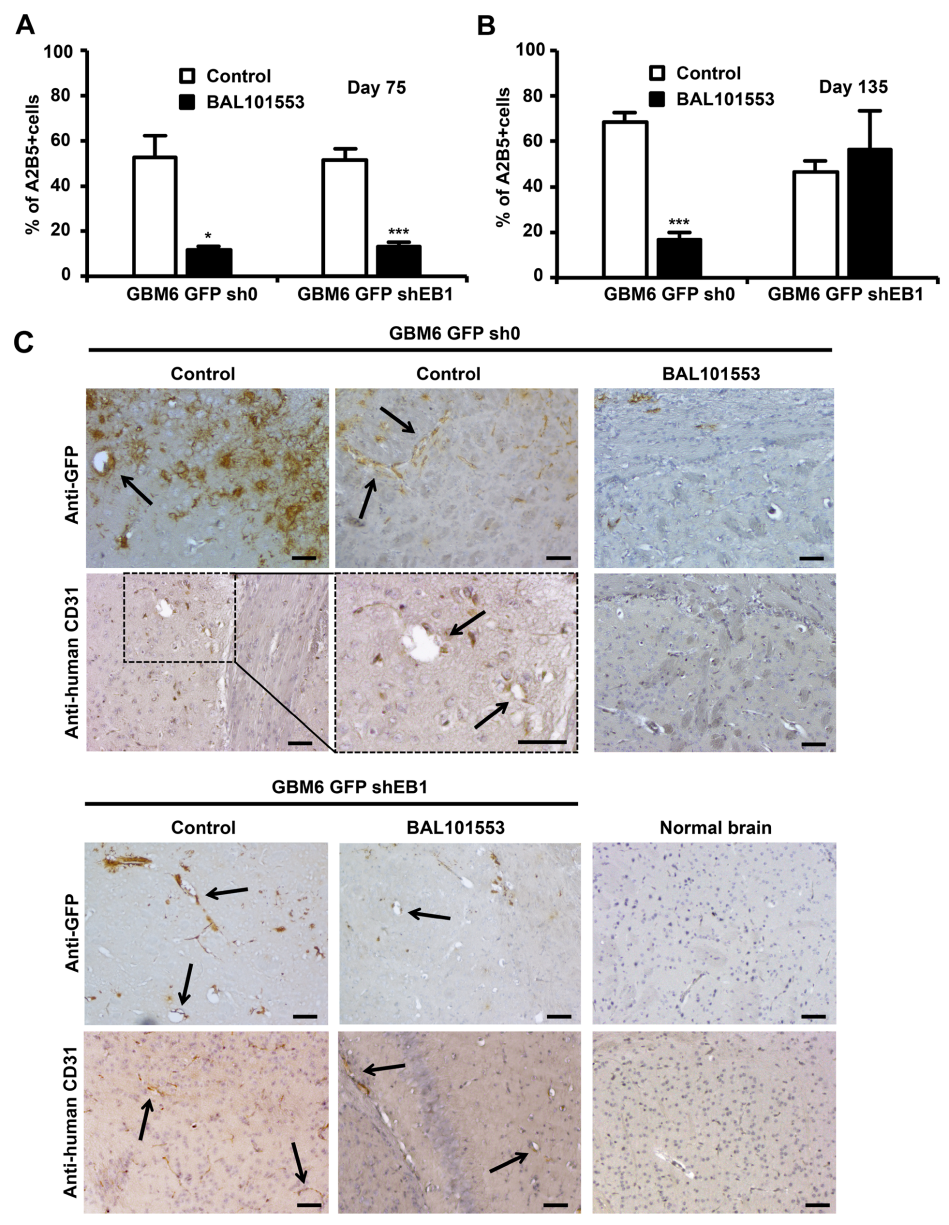
BAL101553 induces loss of stem cell properties and inhibits tumor vasculature Quantification of tumor cells positive for A2B5 staining at 75 **(A)** and 135 days **(B)** post-grafting. **(C)** GFP or human CD31 immunostaining of mice brains grafted or not (normal brains) with GBM6-GFP-sh0 cells or GBM6-GFP-shEB1 cells, at 105 days post-grafting. Arrows show GFP or CD31 positive staining in vascular structures that are only observed in untreated tumors. Bar = 50 µm.

In order to investigate whether BAL101553 could alter stem-cell properties *in vivo*, we first quantified the proportion of CSLCs in tumors under treatment. GFP+ tumor cells were sorted by FACs and tumor cells expressing the stem cell marker A2B5 were quantified (Figure 2A). More than 80% of tumor cells expressed A2B5 at the time of grafting (day 0). At day 75, BAL101553 treatment shifted the proportion of tumor cells in favor of A2B5^-^ cells (-77.8 ± 2.3%; p<0.05 and -74.3 ± 2.6%; p<0,001, for GBM6-GFP-sh0 and GBM6-GFP-shEB1 tumors, respectively (Figure 2A). However, at day 135, the decrease in the proportion of A2B5^+^ cells was only maintained in GBM6-GFP-sh0 tumors (-75.3 ± 4.7%; p<0,001; Figure 2B). These data show that BAL101553 treatment reduced efficiently the proportion of CSLCs in GBM6 orthotopic tumor-bearing mice in EB1-expressing tumors, as long as the drug was administered. To determine whether BAL effect results from a dramatic reduction in tumor volume rather than a specific effect on GBM CSLCs, we then analyzed distribution of tumor CSLCs within brains by immunohistochemistry analysis with an anti-GFP antibody. Importantly, formation of patterned tubule networks by the GBM6-GFP-sh0 cells mimicking endothelial-lined vascular networks was observed inside tumors (Figure 2C). Staining with human anti-CD31 antibody confirmed the presence of vessels containing the human stem cells. Importantly, no staining with anti-GFP and human anti-CD31 antibodies was observed in brain isolated from normal mouse. These results suggest that CSLCs acquire endothelial properties to participate to the tumor vasculature. Following BAL101553 treatment, we have detected in GBM6-shEB1 tumors the presence of several structures stained with anti-GFP and human anti-CD31 antibodies, contrary to GBM6-sh0 tumors, suggesting that the drug blocked CSLCs-acquired endothelial properties in an EB1 expression-level dependent manner.

### *In vitro*, BAL27862 treatment inhibits GBM6 endothelial differentiation, potentiated by EB1 expression

**Figure 3:**
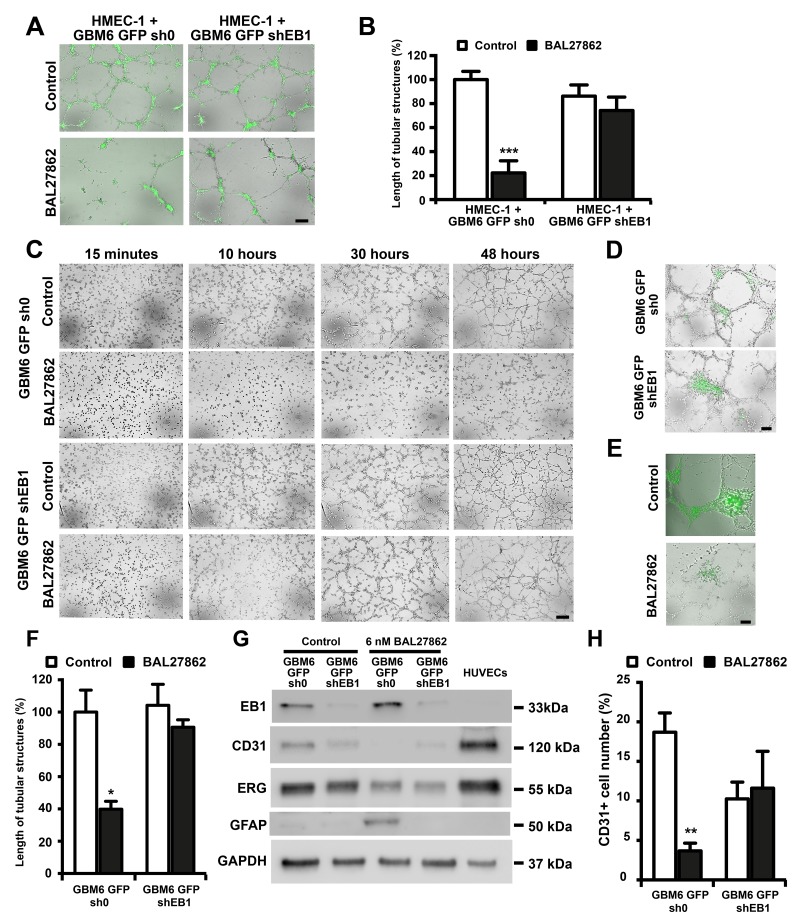
BAL27862 inhibit endothelial differentiation of cancer stem like cells, in an EB1 expression-dependent manner Representative photographs of HMEC-1 cells co-cultured with GBM6-GFP **(A)** or GBM6-GFP cells alone **(C)** on Matrigel Bar = 50 µm. Histogram showing the length of tubular structures in cocultures **(B)** or GBM6-GFP cells alone **(F)**. **(D)** Representative photographs of a preformed HMEC-1 capillary-like network incubated with GBM6-GFP spheres for 48 h. **(E)** Representative photographs of a preformed HMEC-1 capillary-like network co-cultured with GBM6-GFP-sh0 spheres and treated with BAL27862 or vehicle. Bar = 50 µm. **(G)** Western blot analysis of expression of EB1, CD31, ERG and GFAP. GAPDH was used as loading control. **(H)** FACS analysis of CD31 expression in GBM6-GFP cells. Original acquisitions of images from our live-cell imaging analyzer was in the form of quadrants, in order to present the 4 conditions in the same image. However, for a better data presentation in the figure, we have changed the positioning of the 4 images, at each times. For that, we have extracted each image from the quadrant using a rectangular selection, in order to present images vertically in the figure. For GBM6-GFP-sh0 control and GBM6-GFP-shEB1 control (10 hours), we have selected in the same time a little lane of the top of the image just below, explaining the presence of an aberrant lane on the bottom of GBM6-GFP-sh0 control and GBM6-GFP-shEB1 control images in the figure.

We then decided to investigate the potential anti-angiogenic mechanism of the drug on GBM6 cells *in vitro*. We first observed that GBM6 cells could participate to vascular structures under appropriate cell culture conditions *in vitro*. Indeed, GBM6 cells acquired an endothelial phenotype when co-cultured with human endothelial cells, as a typical capillary network including fluorescent cells (GBM6-GFP cells) was observed 48 h after seeding GBM6-GFP-sh0 and GBM6-GFP-shEB1 cells with HMEC-1 on Matrigel (Figure 3A and 3B). To determine whether cell-cell contact between HMEC-1 and GBM6 cells was necessary for tube formation, GBM6-GFP-sh0 and shEB1 were seeded alone on Matrigel. Interestingly, a capillary network was formed 48 h later, suggesting that GBM6 cells can differentiate into endothelial-like cells (Figure 3C and 3F). A favorable microenvironment was required, since GBM6 cells did not form tubes when cultured on a poly-DL-ornithine substrate (Supplementary Figure 2A). No statistically significant difference in the length of tubes was observed between GBM6-GFP-sh0 and shEB1 when co-cultured with HMEC-1 or grown alone, suggesting that the level of expression of EB1 did not influence GBM6 differentiation towards an endothelial phenotype. A difference between control GBM6-GFP-sh0 and GBM6-GFP-shEB1 cells was only observed when spheres of GBM6 were seeded on a preformed vascular network (Figure 3D, Supplementary Video 1). Indeed, GBM6-GFP-sh0 but not GBM6-GFP-shEB1 cells migrated along tubes, confirming the promigratory role of EB1 in CSLCs as previously described [16].

Since GBM6 cells are able to organize into capillary-like tubes in 3D Matrigel cultures, the impact of BAL27862 on the angiogenic potential of GBM6 cells after 48 h was analyzed. A low, non-cytotoxic concentration (6 nM) of BAL27862 (Supplementary Figure 2B) strongly suppressed the formation of capillary-like structures of GBM6-GFP-sh0 when cultured alone (-60.1 ± 4.9 %, p<0.05) or co-cultured with HMEC-1 (-77.8 ± 4.6%, p<0.001) whereas the drug had little impact on GBM6-GFP-shEB1 cells alone (-13.1% ± 4.4%) or co-cultured with HMEC-1 (-14.0% ± 12.9%) (Figure 3A-3C and 3F). Time-course analysis showed that GBM6-sh0 cells did not initiate vessel formation under drug treatment (Figure 3C), suggesting that BAL27862 can inhibit endothelial trans-differentiation of GBM6 cells. However, the low concentration of BAL27862 failed to inhibit the capillary-like network of GBM6-shEB1 cells (Figure 3C and 3F), and HMEC-1 alone (Supplementary Figure 2C and 2E). Moreover, the reduced formation of tubular-like structures was not caused by difference in growth kinetics between control and treated cells, because at 6 nM, BAL27862 had no effect on cell growth (Supplementary Figure 2B and 2D). Furthermore, when GBM6 spheres were deposited on a preformed HMEC-1 capillary-like network, BAL27862 inhibited GBM6-GFP-sh0 colonization on HMEC-1 tubes (Figure 3E). Altogether, these data revealed that sub-cytotoxic concentrations of BAL27862 inhibit the angiogenesis caused by GBM6 CSLCs in an EB1 expression level-dependent manner.

As the endothelial phenotype is characterized by specific biomarkers, the expression of CD31 and ERG in GBM6 cells after BAL27862 treatment was analyzed. Cells were harvested non-enzymatically from Matrigel at the same time as tube length measurement (48 h). GBM6-GFP-sh0 and GBM6-GFP-shEB1 expressed CD31 and ERG, thus confirming endothelial differentiation of GBM6 when cultured on Matrigel (Figure 3G and 3H) but not on poly-DL-ornithine (Supplementary Figure 2F and 2G). Importantly, exposure to a low concentration of BAL27862 (6 nM) triggered a significant decrease in ERG and CD31 expression level in GBM6-GFP-sh0 cells (Figure 3G and 3H). For example, ERG expression was decreased around 3 times as quantified by western blotting and the percentage of CD31-expressing GBM6-GFP-sh0 cells was decreased by 80.3 ± 5.2 % after drug treatment (FACs analysis) (Figure 3H). In addition, an increase in the expression of GFAP was detected in control GBM6-GFP-sh0 cells following drug treatment, which is typical of the astrocytic differentiation (Figure 3G). Altogether, these results show that sub-cytotoxic concentrations of BAL27862 switch the endothelial-like differentiation of GBM6 cells towards an astrocytic phenotype in an EB1 expression-level dependent manner.

### BAL27862 suppressed microtubule dynamic instability

**Figure 4:**
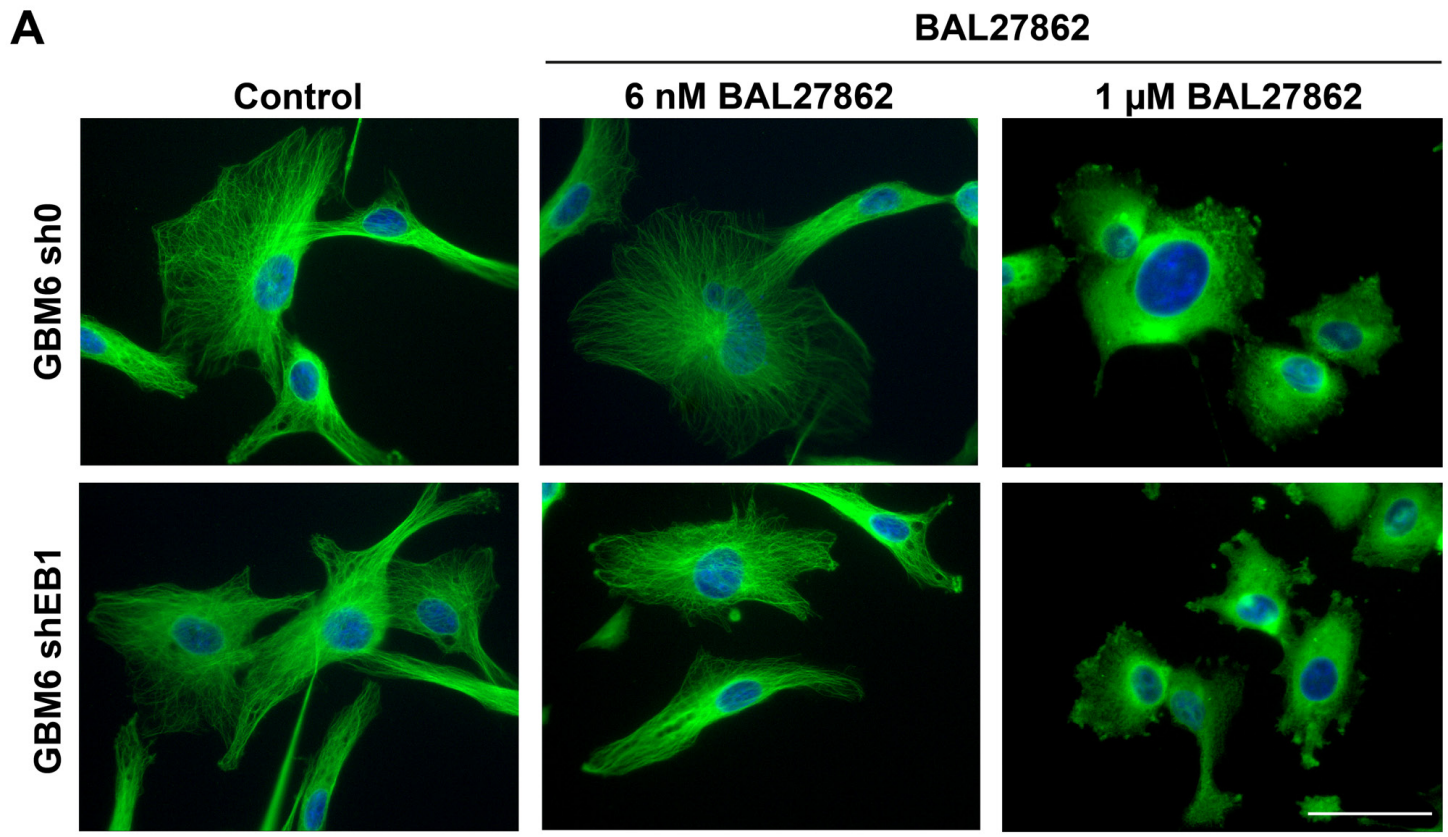
Sub-cytotoxic concentration of BAL27862 does not induce modifications of MT cytoskeleton architecture **(A)** Immunofluorescence staining of α-tubulin in GBM6-sh0 or GBM6-shEB1 treated or not (control) with BAL27862 at 6 nM or 1 µM for 48h. Bar=10 μm.

In order to decipher the mechanism of action of BAL27862 on GBM6 cells, we first performed indirect immunofluorescence of α-tubulin in GBM6-sh0 and GBM6-shEB1 cells. We observed that contrary to high concentration (1 µM), the low concentrations of BAL27862 that inhibited endothelial differentiation (i.e. 6 nM), did not depolymerize MT network in cells suggesting that BAL27862 mainly acts by stabilizing MT dynamics (Figure 4A). We then performed measurements of MT plus-end dynamics, at steady state, in an *in vitro* reconstituted system using dynamic MT and EB3-GFP as a plus-end tracker (Table 2). Nanomolar concentrations of BAL27862 (75-100 nM) suppressed MT dynamics by decreasing the MT growth rate and increasing time and distance-based catastrophe frequencies. Such stabilizing effect of BAL27862 on MT dynamic instability parameters in a reconstituted system is consistent with the effect of several other members of the MTA family, including Taxanes, Epothilones and *Vinca*-alkaloids [21, 22]. In addition, we previously demonstrated the crucial role of MT dynamics alterations by MTA on VEGF signaling disruption in endothelial cells resulting in inhibition of angiogenesis and migration (19, 23), leading us to investigate the role of BAL27862 on VEGF secretion.

**Table 2:** Dynamic instability of microtubules in *in vitro* EB3-GFP tracking assay

Parameters		Control	BAL 27862 75 nM	BAL 27862 100 nM
Number of analyzed MTs		32	32	29
Growth	Rate(µm/min ± SEM)	13.4 ± 0.3	10.9 ± 0.2^**^	8.1 ± 0.2^**^
Catastrophe	min^-1^ ± SE	2.7 ± 0.2	4.3 ± 0.3^**^	5.1 ± 0.5^**^
Catastrophe	μm-^1^ ± SE	0.60 ± 0.03	1.84 ± 0.29^**^	1.00 ± 0.17^*^

^*^p<0.05; ^**^ p<0.01 (vs control)

### BAL27862 inhibits VEGF secretion by GBM6 and human GBM tissue

**Figure 5:**
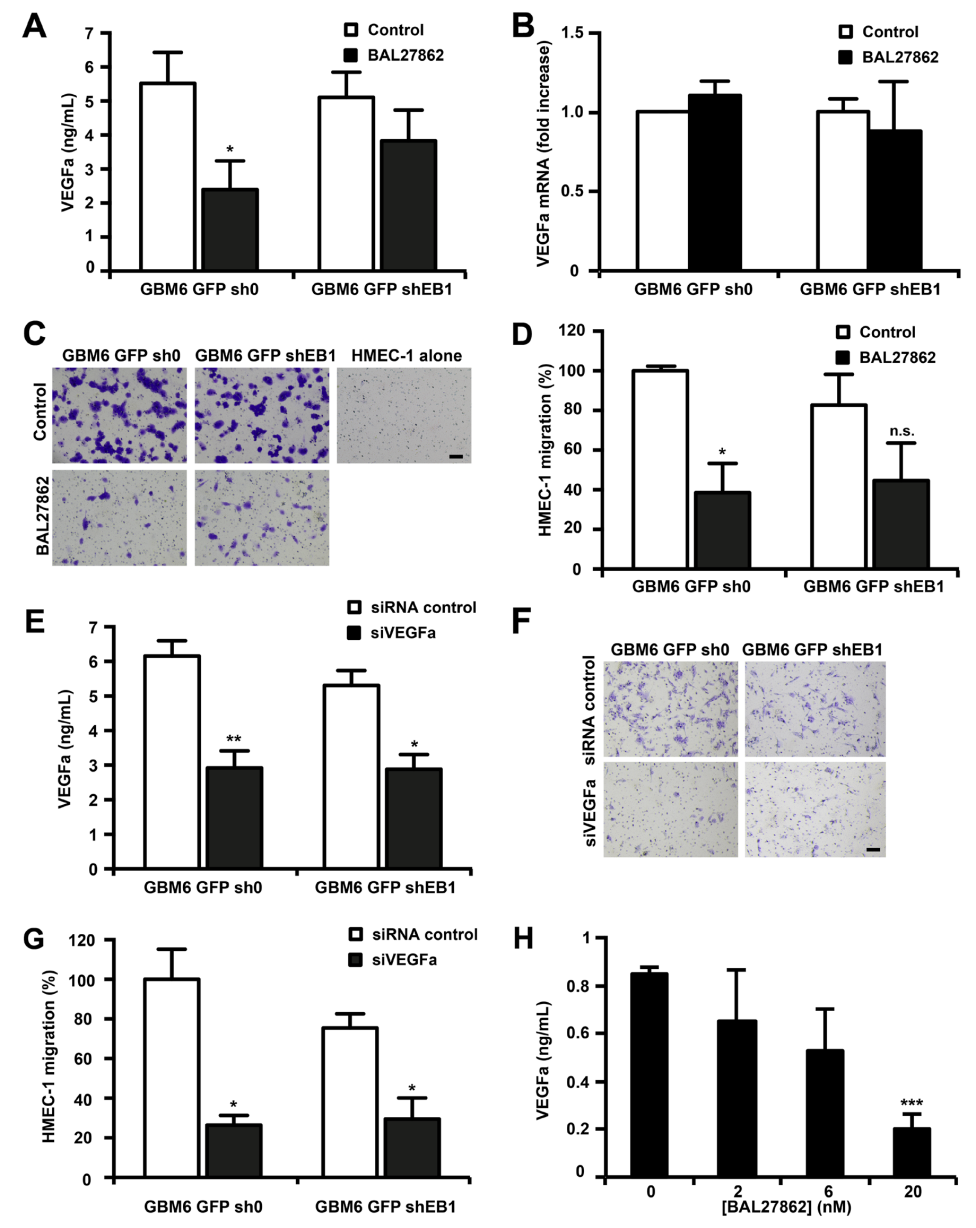
BAL27862 inhibits VEGF secretion by GBM6 cells and GBM6-induced migration of endothelial cells **(A)** VEGFa protein level measurement in cell culture supernatants of GBM6-GFP cells. **(B)** mRNA levels of VEGFa analyzed by quantitative RT-PCR. **(C)** HMEC-1 cell migration induced by GBM6-GFP cells. Bar = 200 μm. **(D)** Quantification of migratory HMEC-1 cells, expressed as percentage of migrating cells relative to 100% of control GBM6-GFP-sh0 cells. **(E)** VEGFa protein level measurement in cell culture supernatants of GBM6-GFP cells transfected with siRNA against VEGFa (siVEGFa) or siRNA control.**(F)** HMEC-1 cell migration induced by GBM6-GFP cells transfected with siVEGF or siRNA control. Bar = 200 μm. **(G)** Quantification of migratory HMEC-1 cells, expressed as percentage of migrating cells relative to 100% of control GBM6-GFP-sh0 cells. **(H)** VEGFa protein level measurement in cell culture supernatants of GBM tissue explants.

Besides differentiation of CSLCs to become part of the tumor vasculature as endothelial cells, CSLCs can also exert paracrine effects on endothelial cell by secreting soluble factors to stimulate tumor angiogenesis [13, 14]. To examine this, we employed an *in vitro* model of angiogenesis in which HMEC-1 cells were induced to migrate when stimulated by angiogenic factors [24] (Supplementary Figure 3). First, we measured the concentration of VEGF in the culture medium of GBM6-GFP-sh0 and shEB1 grown on Matrigel in the lower chamber after BAL27862 treatment. As shown in Figure 5A, a low, non-cytotoxic concentration of BAL27862 inhibited secretion of VEGF by 57 ± 15% (p<0.05) and 25 ± 18% in GBM6-GFP-sh0 and GBM6-GFP-shEB1 cells, respectively. However, mRNA levels of VEGF were not altered by BAL27862 (Figure 5B). Then an upper chamber containing HMEC-1 cells was inserted and the percentage of HMEC-1 cells migrating to the lower chamber containing GBM6 cells was quantified. In experiments with vehicle-treated GBM6 cells, HMEC-1 cells triggered pronounced migration, while the increased migration of HMEC-1 cells was significantly prevented when GBM6 cells were pretreated with BAL27862 (Figure 5C and 5D). In control experiments where GBM6 were not seeded in the lower chamber, HMEC-1 cells were unable to migrate, meaning that a soluble factor released by GBM6 was necessary for migration of endothelial cells (Figure 5C). When GBM6 were cultured on poly-DL-ornithine, no VEGF was secreted and no migrating HMEC-1 cell was scored (Supplementary Figure 4A-4C). Finally, we confirm that VEGF secretion by GBM6 cells was involved in endothelial cell migration by using siRNA to deplete VEGF in GBM6-GFP-sh0 and shEB1 seeded on the lower chamber (Figure 5E). As shown in Figures 5F and 5G, the number of HMEC-1 cells migrating to the lower chamber was decreased after VEGF down-regulation (-77.3 ± 4.9%; p<0.05 and -60.8 ± 14.0%; p<0.05, for GBM6-GFP-sh0 and GBM6-GFP-shEB1 cells, respectively). Altogether, these results reveal that sub-cytotoxic concentrations of BAL27862 inhibit VEGF secretion by GBM6 cells and consequently suppress GBM6-induced migration of endothelial cells. These effects were lower in EB1-down regulated stem cells. Finally, BAL27862 decreased VEGF secretion in a human GBM explant culture after 3 days of exposure, as shown *in vitro* with GBM6 (Figure 5H).

## DISCUSSION

In this study, we have demonstrated that long-term daily, oral treatment with BAL101553 strongly enhanced survival of mice when orthopically grafted with human GBM CSLCs. Indeed, a 100 day-treatment was sufficient to provoke a survival benefit that was almost one year longer than observed in vehicle-treated mice. This result emphasizes our previous work showing that a short-term treatment with BAL101553 administered intravenously (on days 30, 33 and 36 after tumor implantation) induced a moderate EB1-dependent survival benefit in tumor-bearing mice. BAL101553 activity was higher in control CSLCs than in EB1 down-regulated CSLCs, thus confirming the role of EB1 expression as a potential biomarker for drug response [16, 19]. Post-mortem analysis of brains indicated that tumor growth was reduced and tumor spreading was limited under treatment, while it invaded the surrounding parenchyma in vehicle-treated mice. However, the tumor was not totally eradicated suggesting that additional treatment in a maintenance therapy schedule may have maintained a higher level of tumor growth inhibition; perhaps, with an intermittent schedule to limit resistant cell emergence [25]. The good tolerability and oral bioavailability of BAL101553 are important advantages when considering long-term treatments.

Our study shows for the first time that BAL101553 exerts anti-angiogenic effects *in vivo* and *in vitro* by acting on GBM CSLCs. Furthermore, as also reported for other GBM stem models, we demonstrated that GBM6 cells have the ability to activate the angiogenic switch; a critical transition point for tumor growth [26-28]. To decipher the anti-angiogenic mechanism of the drug, experiments were designed on Matrigel to mimic *in vitro* the process of CSLC trans-differentiation into endothelial cells in particular microenvironmental conditions [9]. Cell cultures on poly-D-ornithine were conducted as negative controls. BAL27862 treatment caused a switch of GBM6 endothelial differentiation towards an astroglial phenotype, consistent with our previous observations [16]. Furthermore, it altered the contribution of CSLCs to normal endothelial cell behavior by decreasing VEGF secretion by GBM6 cells and, thereby, reducing endothelial cell migration and tube formation. A decrease in VEGF secretion induced by the drug was also observed in human GBM explants that contain around 30% of cancer stem cells. This point is of interest, as VEGF secretion is known to initiate angiogenesis [29]. Our results confirm the angiogenic dependency of GBM [30]; however, anti-angiogenic therapeutics were unsuccessful so far. The administration of bevacizumab, an anti-VEGFa antibody, to GBM patients improved progression-free survival but failed to show a survival benefit, because many patients rapidly developed resistance [31]. The resistance mechanism(s) is not yet elucidated. The ability of CSLCs to trans-differentiate into endothelial cells that display low sensitivity to bevacizumab or other anti-VEGFR therapies has been suggested [32, 33]. Inhibition of the endothelial trans-differentiation process of CSLCs by BAL101553 could potentially overcome anti-angiogenic therapy resistance in GBM, by acting upstream in the signaling cascade of angiogenesis, as compared with the anti-angiogenic drugs currently marketed. Several MTA, such as *Vinca*-alkaloids and combretastatin A4 and its derivatives have been described for antiangiogenic and antivascular properties by inhibiting the formation of endothelial capillary –like structures and disrupting preformed tubular structures from endothelial cells [34-36]. BAL 27862 shares these properties. Importantly, our study is the first showing that suppressing MT dynamics leads to reduction in VEGF secretion, which mechanism remains to decipher but probably impact VEGF trafficking as no effect was observed on mRNA levels. Furthemore, BAL27862 exerts anti-angiogenic activity by tackling CSLCs, such effect was observed at low concentrations that were not cytotoxic and did not alter vessel formation by normal endothelial cells. Moreover, the dose used for *in vivo* experiments was well-tolerated, and no sign of vascular toxicity such as hemorrhage was detected.

Our results highlight EB1-dependent effects of BAL101553, which were observed mostly in control sh0 cells, suggesting that the mechanism of sensitization to BAL101553 by EB1 in angiogenesis is related to stabilization of MT dynamics. Through its C-terminal part, EB1 interacts with numerous partners at growing MT ends to form molecular networks that contribute to the regulation of MT dynamic instability and regulate MT functions [37]. In addition, the amount of EB1 influences the way MT dynamic instability is altered [38] and post-translational modifications of EB1 also modulate the regulation of MT dynamics, proliferation /migration and subsequent MTA response [23, 38, 39]. Interestingly, VEGF suppressed MT dynamics in living HUVECs, induced EB1 C-terminal detyrosination and increased EB1 comet length. Vinflunine, a MTA of the *Vinca*-alkaloid family, decreased the level of the detyrosinated form of the C-terminal of EB1, increased the native tyrosinated form of EB1 and completely abolished the effect of VEGF on EB1 comets [23]. Vasohibins were identified as intrinsic factors in endothelial cells with some inhibitory effect on angiogenesis [40, 41]. However, they are overexpressed upon VEGF treatment of endothelial cells, suggesting that they have a more complex role in angiogenesis. Interestingly, vasohibins have been recently found as the first enzymes able to detyrosinate the C-terminal EEY sequence of tubulin and EB1 [42]. All these new findings highlight questions on the functionality of the tyrosination cycle of tubulin and EB1 in cancer stem cells and the role of cytoskeleton and drugs targeting MT in angiogenesis. Moreover, it has been recently proposed that the loss of Golgi-MT attachment resulting from EB binding to MT minus end in mutant EB1/EB3 cells might affect cell polarity and thus cell motility in 3D matrices [43]. Functional consequences of EB1 binding to the minus end of MT remain to be understood.

Altogether, these results show that BAL101553 counteracts the formation of brain tumor vessels by inhibiting GBM stem cell trans-differentiation in tumor-derived endothelial cells, as well as VEGF secretion; thus, cutting off the tumor from blood and nutrition supply and potentially adding to the direct anti-tumor cell effect. A high level of EB1 expression in CSLCs potentiates the drug effects, further supporting the potential of EB1 expression as a BAL101553 response-predictive biomarker in GBM.

## MATERIALs AND METHODS

### Compounds

BAL27862 and BAL101553 were provided by Basilea Pharmaceutica International Ltd (Basel, Switzerland). BAL27862 is the active moiety of the lysine prodrug BAL101553, which was developed to improve solubility without the need of excipients associated with side effects. Hence, the prodrug BAL101553 was used for all animal experiments. As the cleavage of the prodrug BAL101553 is incomplete in cell culture medium, the active moiety BAL27862 was used for *in vitro* assessments.

### Cell lines and cell culture

GBM6 clones were previously obtained after stable transfection of the GBM stem-like cell line GBM6; the GBM6-shEB1 and GBM6-GFP-shEB1 clones deficient for EB1 and the negative shRNA control cell lines GBM6-sh0 or GBM6-GFP-sh0 [16]. When necessary, cells were seeded on 10 μg/mL poly-DL-ornithine to allow adherance without differentiation [16]. Human umbilical vein endothelial cells (HUVECs) and the human dermal microvascular endothelial cell line (HMEC-1) were obtained from the Cell Culture Laboratory at Assistance Publique-Hôpitaux de Marseille. HMEC-1 were grown in MCDB-131 medium containing 10% heat-inactivated fetal bovine serum, 2 mmol/L glutamine, 1% penicillin, and streptomycin (all from Life Technologies), 1 μg/mL hydrocortisone (Pharmacia & Upjohn), and 10 ng/mL epithelial growth factor (R&D Systems). HUVECs were grown in RPMI 1640 medium (Life Technologies) containing 20% heat-inactivated fetal bovine serum, 2 mmol/L glutamine, 1% penicillin, and streptomycin, 50 IU/mL sodium heparin (Sanofi-Synthelabo), and 50 μg/mL endothelial cell growth supplement (BD Biosciences). For VEGFa silencing by transient transfection, cells were transfected by lipofectamine 2000 system with siRNA for VEGFa (Silencer Select siRNAs s228861, Thermo Fisher Scientific, NY, USA and Silencer Select negative control, Thermo Fisher Scientific). VEGFa down-regulation was analyzed 96 h later using an ELISA kit (Sigma-Aldrich).

### Explant culture of human glioblastoma tissue

GBM tissue samples from a GBM patient were collected after surgery and placed in Dulbecco's modified Eagle's medium (DMEM) supplemented with 0.5% fetal calf serum (FCS), 1% penicillin-streptomycin and 1% sodium pyruvate (Gibco-Invitrogen, Cergy Pontoise) [44]. Tissues were cut into 500 μm pieces in defined serum-free stem cell medium [16] and plated on 12-well plates precoated with poly-(L)-lysine (10 μg/mL; Sigma). Medium was supplemented with 0.4% methycellulose (Sigma). Explants cultures were then incubated at 37°C in a humidified atmosphere of 5% CO2. After 72 h of culture, explants were treated with 2, 6 or 20 nM of BAL27862. After 72 h of treatment, explants were processed for analysis of VEGFa protein secretion using an ELISA kit (Sigma-Aldrich).

### Cytotoxic assay

Cells (5000 cells/well) were seeded on poly-DL-ornithine (Sigma-Aldrich) coated 96-well plates (10 µg/mL) and allowed to grow for 24 h before treatment with BAL27862. Growth inhibition of cells was measured after 72 h by using a sulforhodamine B assay kit (Sigma-Aldrich) as described previously [19].

### Western blot analysis

GBM6 cells (3×10^5^) were seeded onto 6-well plates previously coated with Matrigel or poly-DL-ornithine and incubated with BAL27862 or vehicle for 48 h. Cells were then harvested and lysed as previously described [16]. Thirty µg of total protein lysate were resolved using a 12% SDS-PAGE gel and blotted onto a nitrocellulose membrane (Bio-Rad laboratories). The membrane was blocked with 5% milk (powder) in Phosphate Buffer Saline (PBS) (Life technologies)-Tween (Sigma-Aldrich) pH 7.4 for 1 h and then incubated in PBS-Tween 0.1%-5% milk solution with mouse anti-EB1 antibody (clone 5, BD Biosciences), rabbit α-GFAP (Abcam), rabbit α-CD31 (Abcam), rabbit α-ERG (Abcam) or mouse α-GAPDH (Sigma-Aldrich). After washing, membranes were incubated with anti-mouse peroxydase-conjugated secondary antibodies (Jackson Immunoresearch) for 1 h. The bound antibodies were then detected using a chemiluminescence detection kit (Millipore). Signals were recorded with G:BOX (Syngene/Ozyme) and quantified with Image J software.

### *In vitro* FACS analysis

GBM6 cells were seeded onto 6-well plates previously coated with Matrigel or poly-DL-ornithine and incubated with BAL27862 or vehicle for 48 h. Cells were then harvested and incubated with CD31-PE antibody (clone AC128, reference 130-092-653) (Miltenyi Biotec). Cells were fixed and analyzed using flow cytometry (FACS Calibur™, BD Biosciences). A total of 100,000 events were counted for each sample and data were recorded with CellQuest Pro Software (BD Biosciences) and analyzed using FlowJo software (Tree Star Inc., San Carlos, CA) choosing the Dean-Jet-Fox model analysis.

### Endothelial cell capillary-like tube formation assay

Matrigel (Corning) was thawed at 4°C and 48-well plates were coated with Matrigel, which was then allowed to solidify for 1 h at 37°C before cell seeding. Cells (3x10^4^ GBM6 or HMEC-1 alone, or 15x10^3^ GBM6 with 15x10^3^ HMEC-1 for co-culture experiments) were then added in medium with BAL27862 or vehicle. For analysis of GBM6 invasion on a well-organized capillary-like network, HMEC-1 were first seeded on Matrigel and incubated without BAL27862 or vehicle for 15 h, then 10 GBM6 spheres were added concomitantly with BAL27862 or vehicle. Cells were allowed to undergo morphogenesis and form capillary-like structures and photographs were taken at indicated time points using the 4X objective of a Juli^TM^ Stage live-cell imaging analyzer (NanoEnTek), and the total length of capillary tubes formed was measured using Image-Pro Plus software.

In order to analyze the potential changes in protein expression, GBM6 cells (3×10^5^) were seeded onto 6-well plates previously coated with Matrigel and harvested at 48 h. For western blot analysis, cells were incubated with a Cell Recovery Solution (BD Biosciences) for 1 h at 4°C under agitation to allow complete dissolution of the Matrigel, then pelleted, washed with cold PBS and finally lysed as described in the western blotting section. For FACs analysis, cells were incubated with 100 units/well of Dispase (Corning) for 2 h at 37°C under agitation to allow complete dissolution of the Matrigel, then pelleted and washed with PBS.

### Transwell migration

To assay GBM6–induced migration of endothelial cells, a co-culture assay using migration chambers as described by Tsujii et al was used [24]. Assays were performed in 24-well transwell chambers (8.0 μm diameter pore). GBM6 cells (6x10^4^) were exposed to BAL27862 or vehicle (control) for 48 h on Matrigel. For VEGFa down-regulation experiments, cells were seeded 24 h after siRNA transfection. After treatment, the medium from the lower chamber was replaced with fresh medium without BAL27862 or vehicle, and the VEGFa protein released was determined using an ELISA kit (Sigma-Aldrich). Alternatively, an insert containing HMEC-1 cells (5x10^4^) in MCDB-131 medium without SVF was then placed into the lower chamber. After 24 h, cell migration was quantified as described [19].

### Indirect immunofluorescence analysis

GBM6 cells were grown on 8-well chamber slides (Labtek, Thermo Fisher Scientific), precoated for 1 h with poly-DL-ornithine (Sigma-Aldrich) (10µg/ml), to be treated for 48 h with BAL27862. As previously described [19], cells were incubated with an anti- α-tubulin (clone DM1A; Sigma-Aldrich) primary antibody, and then with Alexa488 secondary antibody (Invitrogen). Staining was observed using either a Leica DM-IRBE microscope. Images were acquired using Metamoph software and were processed using Image J software.

### *In vitro* plus end tracking assay of GFP-EB3 and measurements of MT dynamics:

The assay was performed by growing MTs in the presence of 13 μM purified tubulin (Cytoskeleton, Denver, CO) from GMPCPP-stabilized (Sigma–Aldrich, St. Louis, MO) and paclitaxel-stabilized MT seeds, which were immobilized on glass coverslips using Poly-l-Lysine PEG-biotin (SuSoS, Dübendorf, CH)–streptavidin (Invitrogen, Carlsbad, CA, Lonza, Basel, CH) links, as described in [45]. BAL 27862 was added at the beginning of MT polymerization.

### RNA extraction

Total RNA was extracted using QIAamp® RNA Blot Mini kit (Qiagen, Courtaboeuf, France) according to the manufacturer’s instructions. RNA samples with no evidence of ribosomal peak degradation and RIN values of 6–10 were used for real-time quantitative PCR analyses [46] after treatment with 1U ribonuclease-free deoxyribonuclease (Roche Applied Science, Meylan, France) at 37°C for 15 minutes.

### Real-time quantitative PCR

RNA samples were processed using a LightCycler 480 instrument (Roche Applied Science) and a LightCycler 480 SYBR Green I Master Mix (Roche Applied Science). Briefly, total DNA-free RNA (1 μg) was reverse-transcribed into cDNA using 1 μg of random hexamers and Superscript II reverse transcriptase as recommended by the manufacturer (Invitrogen Life Technologies, Cergy Pontoise, France). Measurements were performed in triplicate for each sample, and relative expression ratios of target gene transcripts (VEGFa) and reference gene transcripts (18S, GAPDH) were calculated using qPCR efficiencies and cycle threshold (Ct) deviations [47]. RNA expression levels in GBM CSLCs were subsequently expressed as percentages compared to GBM6 GFP sh0 control cells corresponding to 100% of expression. Sequence details for both forward and reverse primers are as follows: forward primer 18S: 5′-CTACCACATCCAAGGAAGGCA-3′; reverse primer 18S: 5′- TTTTTCGTCACTACCTCCCCG-3′; forward primer GAPDH: 5′- CAAATTCCATGGCACCGTC-3′; reverse primer GAPDH: 5′- CCCACTTGAT-TTTGGAGGGA-3′; forward primer VEGFa: 5′-AGGAGGAGG GCAGAATCA-3′; reverse primer VEGFa: 5′- AGGGTCTCGATTGGATGGC-3′. PCR conditions were as follows: 10 min at 95°C, followed by 35 cycles of 15 s at 95°C, 30 s at 67°C for 18S or 45 cycles of 15 s at 95°C, 30 s at 65°C for GAPDH or 30 s at 66°C for VEGFa.

### Animal experiments

All experimental procedures and animal care were carried out in conformity with the guidelines of the French Government and approved by the Regional Committee for Ethics on Animal Experiments (authorization number 0100903). Six to 8 weeks old female athymic nude mice were obtained from Harlan Laboratories France and stereotactically grafted with GBM6-GFP-sh0 or shEB1 cells as previously described [16]. Four groups (n=8) were randomized for GBM6-GFP-sh0- and GBM6-GFP-shEB1-bearing mice respectively, receiving daily oral administrations of BAL101553 (30 mg/kg in 10 ml/kg application volume) or vehicle solution (10 ml/kg, control groups) from day 35-135 after glioma implantation (5 administrations / week). Animals were monitored each day for weight loss, ataxia, and periorbital hemorrhage. Animals were euthanized when affected by hemiplegia or 20% weight loss. At 75, 105 and 135 days after glioma implantation, three animals of each group were sacrificed for analysis of tumor volumes by 3D-reconstruction, for FACS analysis [16] and for immunohistochemistry staining.

### Immunohistochemistry

GFP or CD31 immunohistochemistry was carried out in tumor area, using an anti-GFP primary antibody (Abcam) or an anti-human CD31 primary antibody (clone JC/70A) (Dako) and avidin–biotin–peroxidase method. Isotype control antibody (BD Biosciences) was used at the same concentration as primary antibody. Brains from non-grafted mice were used as control.

### 3D reconstruction

For each brain, a complete collection of serial cryo-sections was imaged by automatic scanning microscope and processed by For3D as described [48, 49] using a Miraxmidi slide scanner (Zeiss, Jena, Germany). ImageJ and homemade Matlab functions were used to render brains and tumors in 3D. Autofluorescence was used to detect and segment the whole brain tissue. GBM cells expressing GFP were segmented from autofluorescence by subtracting background in Image J and applying higher threshold values. Matlab was used to identify individual volumes of the 3D GBM tumors within each brain, as described [48, 49].

### Statistical analysis

Data are presented as mean ± S.E.M. At least, three independent experiments were performed for each condition in cellular studies. *In vitro* data were analyzed using the two-tailed Student’s t test. Survival medians were estimated by the Kaplan-Meier product limit method. The log-rank test was used to compare survival rates by univariate analysis. Reported p-values are two-sided, and p<0.05 was considered statistically significant. Asterisks indicate significant level vs control ^*^, p < 0.05; ^**^, p < 0.005; ^***^, p < 0.001. Statistical analyses were performed with GraphPad 5.0 statistical software.

## SUPPLEMENTARY MATERIALS FIGURES AND VIDEO




