# Supplementary material for: EB1-dependent long survival of glioblastoma-grafted mice with the oral tubulin-binder BAL101553 is associated with inhibition of tumor angiogenesis
Source: Oncotarget. 2020 Feb 25;11(8):759–74. doi: 10.18632/oncotarget.27374 (PMC7055546; doi:10.18632/oncotarget.27374)
Supplement: Supplementary file 1 [file oncotarget-11-759-s001.pdf]

EB1-dependent long survival of glioblastoma-grafted mice with the oral tubulin-binder BAL101553 is associated with inhibition of tumor angiogenesis

SUPPMENTARY MATERIALS

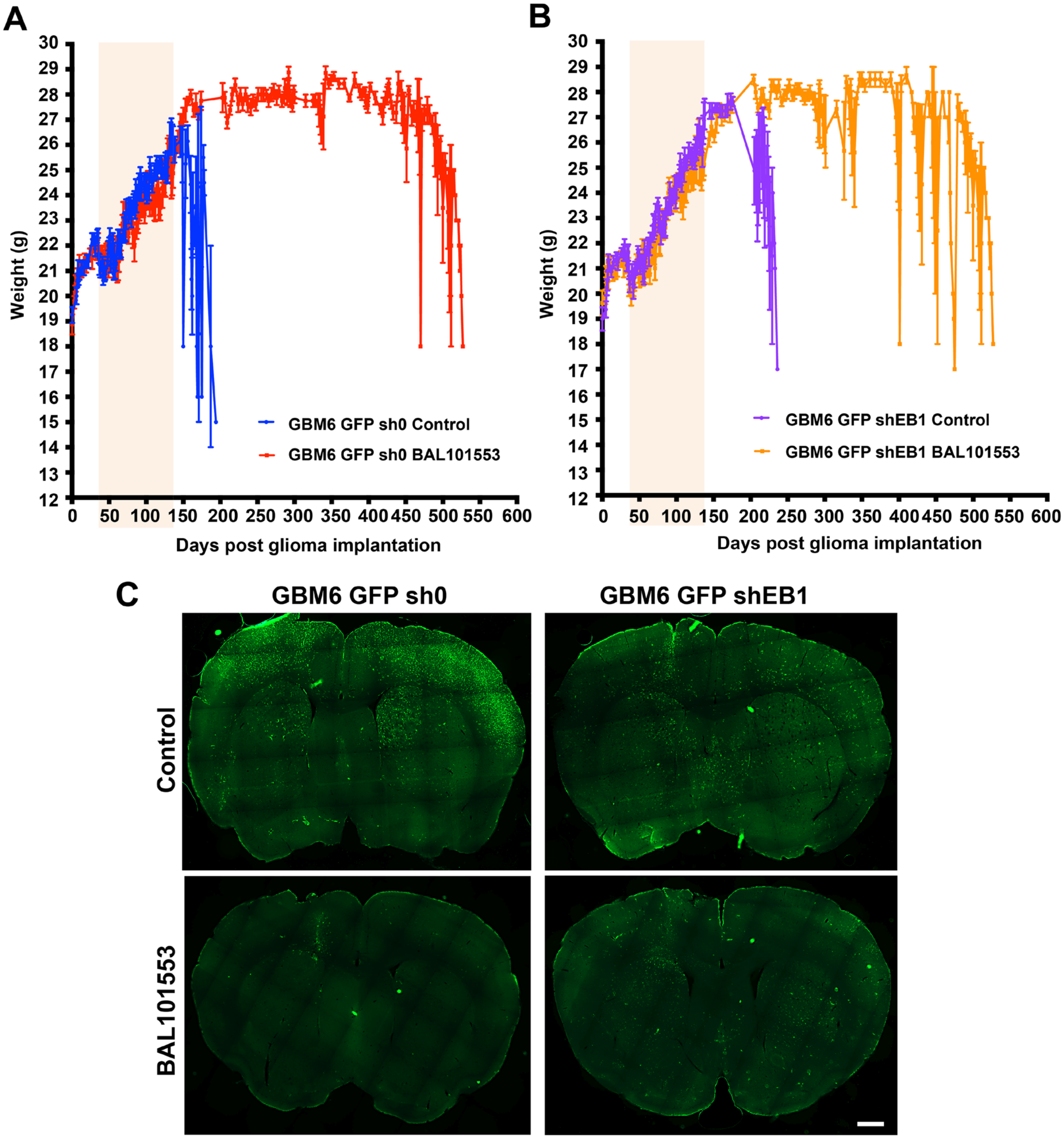

Supplementary Figure 1: Mean weights of GBM6-GFP-sh0 **(A)** GBM6-GFP-shEB1 bearing mice **(B). (C)** Sagittal sections show GBM6-GFP-sh0 or GBM6-GFP-shEB1 tumor cells (GFP green fluorescence) in brain of mice treated with BAL101553 or vehicle Bar = 1000 µm.

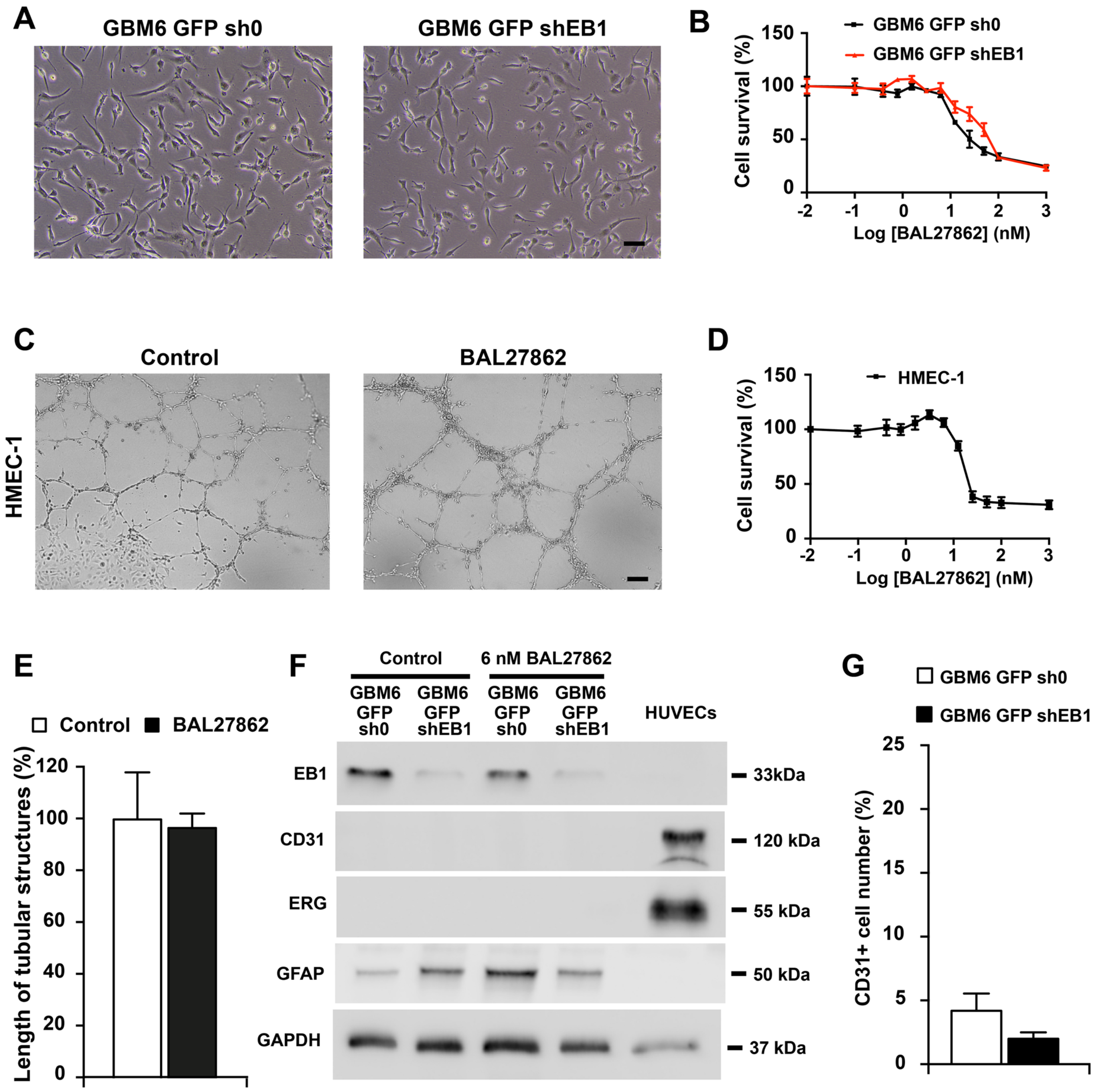

Supplementary Figure 2: (A) Representative photographs of GBM6-GFP cells seeded on poly-DL-ornithine Bar = 100 µm. **(B)** Dose response curves of the cytotoxicity of BAL27862 in GBM6-GFP-sh0 or GBM6-GFOP-shEB1 cells. **(C)** Representative photographs of HMEC-1 cells incubated on Matrigel and concomitantly treated with BAL27862 or vehicle Bar = 50 µm. **(D)** Dose response curves of the cytotoxicity of BAL27862 in HMEC-1 cells. **(E)** Histogram showing the length of tubular structures in HMEC-1 cells. **(F)** Western blot analysis of expression of EB1, CD31, ERG and GFAP in GBM6-GFP cells cultured on poly-DL-ornithine. GAPDH was used as loading control. **(G)** FACS analysis of expression of CD31 cultured on poly-DL-ornithine.

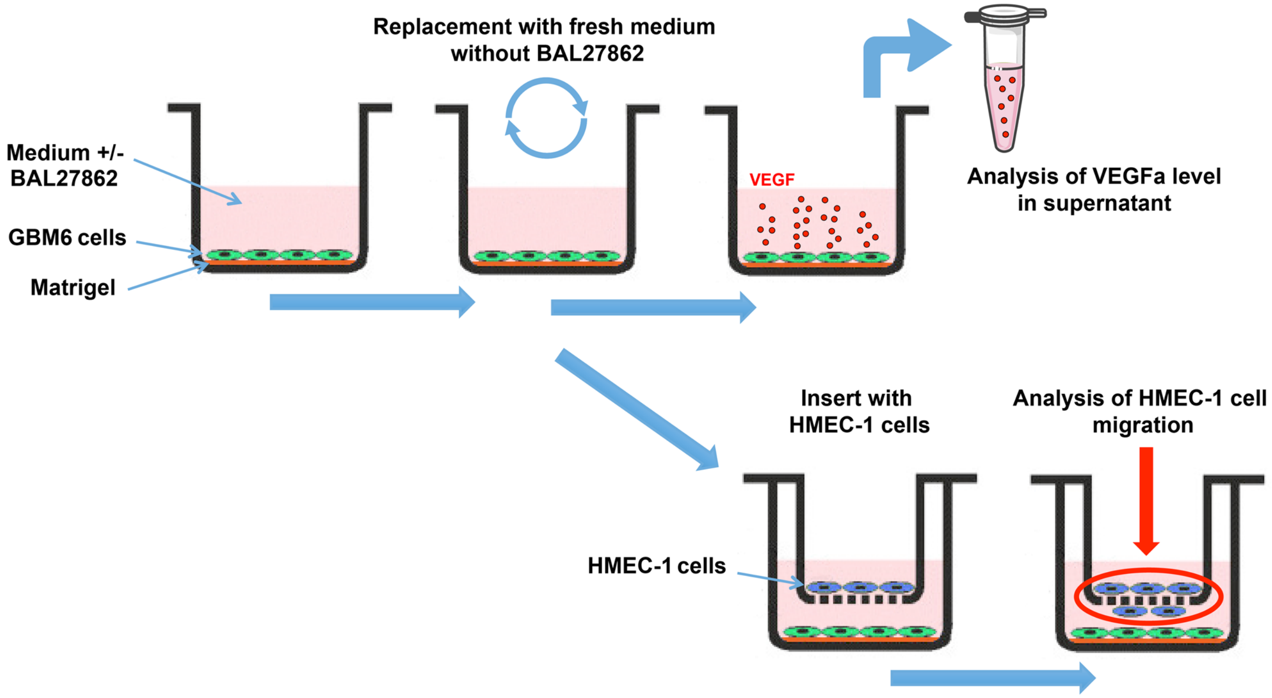

Supplementary Figure 3: Schematic diagram of the VEGFa level assay and GBM6-induced migration assay of endothelial cells.

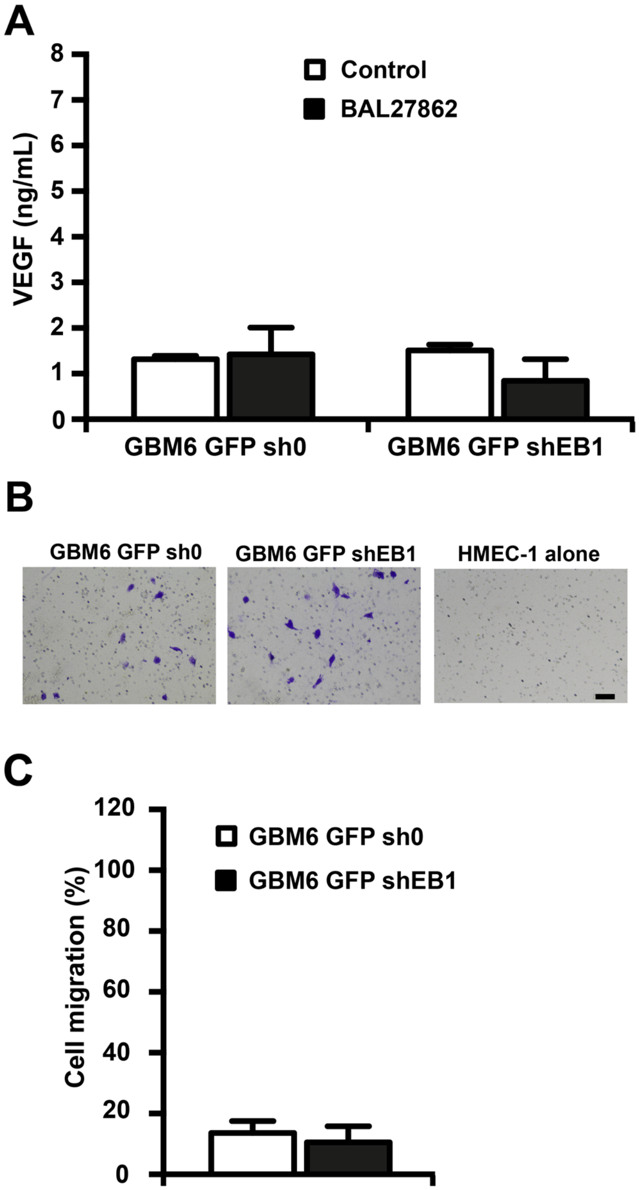

Supplementary Figure 4: (A) VEGF protein level measurement in cell culture supernatants of GBM6-GFP cells seeded on poly-DL-ornithine. **(B)** HMEC-1 cell migration induced by GBM6-GFP cells seeded on poly-DL-ornithine Bar = 200 µm. **(C)** Quantification of migratory cells, expressed as percentage of migrating cells relative to 100% of control GBM6-GFP-sh0 cells.

Supplementary Video 1: Time-lapse imaging illustrating that EB1 is required for migration of GBM6 on HMEC preformed vessels. GBM6-GFP-sh0 (top panels) or GBM6-GFP-shEB1 spheres (bottom panels) were deposited on a preformed HMEC-1 capillary-like network and incubated for 48 h. Cells were time-lapse recorded at 32-min intervals over 48 h period under a phase-contrast and fluorescence microscope using 4X objective. Merged images and time-lapse movie (MPEG-4, 958x720, 7.1 Mo) were obtained using of a JuliTM Stage live-cell imaging analyzer (NanoEnTek).

See Supplementary Video 1
